# Comparison of *in vivo* 3D cone-beam computed tomography tooth volume measurement protocols

**DOI:** 10.1186/s40510-014-0069-2

**Published:** 2014-12-23

**Authors:** Darren Forst, Simrit Nijjar, Carlos Flores-Mir, Jason Carey, Marc Secanell, Manuel Lagravere

**Affiliations:** Department of Dentistry, University of Alberta, Edmonton Clinic Health Academy, 5th Floor, 11405 - 87 Avenue NW, Edmonton, Alberta T6G 1C9 Canada; Faculty of Dentistry – Department of Preventive Dental Science, D341 Dental Building, 790 Bannatyne Avenue, Winnipeg, Manitoba R3E 0W2 Canada; Department of Mechanical Engineering, University of Alberta, 4-9 Mechanical Engineering Building, Edmonton, Alberta T6G 2G8 Canada

**Keywords:** Cone-beam computed tomography, Tooth, Imaging, Three-dimensional

## Abstract

**Background:**

The objective of this study is to analyze a set of previously developed and proposed image segmentation protocols for precision in both intra- and inter-rater reliability for *in vivo* tooth volume measurements using cone-beam computed tomography (CBCT) images.

**Methods:**

Six 3D volume segmentation procedures were proposed and tested for intra- and inter-rater reliability to quantify maxillary first molar volumes. Ten randomly selected maxillary first molars were measured *in* vivo in random order three times with 10 days separation between measurements. Intra- and inter-rater agreement for all segmentation procedures was attained using intra-class correlation coefficient (ICC).

**Results:**

The highest precision was for automated thresholding with manual refinements.

**Conclusions:**

A tooth volume measurement protocol for CBCT images employing automated segmentation with manual human refinement on a 2D slice-by-slice basis in all three planes of space possessed excellent intra- and inter-rater reliability. Three-dimensional volume measurements of the entire tooth structure are more precise than 3D volume measurements of only the dental roots apical to the cemento-enamel junction (CEJ).

## Background

Historically, the *in vivo* detection of changes to dental root morphology such as those associated with external root resorption (ERR) during the course of orthodontic treatment or related to trauma has been mainly through use of two-dimensional (2D) radiographs, most notably periapical radiographs [1-3]. Although histological studies have identified a relatively high incidence of apical ERR, 2D radiographic studies have been less definitive [4,5] and have in general proven inaccurate for the reliable detection of small ERR defects [6]. In fact, 2D periapical radiographs do not reveal external root resorption to an appreciable extent, except for frank apical root resorption, which appears to be in their realm of identification [7]. In addition, there are geometric limitations associated with 2D imaging of a three-dimensional (3D) phenomenon; therefore, the quantitative value of 2D radiographs to measure ERR is questionable [8-10]. When considering panoramic films, the distortion in both tooth position and angulation combined with varying magnification, distortion, superimposition, and imaging artifacts in different parts of the image [11,12] leads to similar limitations in the use of panoramic films to assess ERR [13-15]. Therefore, although 2D radiography may be a good screening tool, its use in the quantification of ERR remains controversial [9].

Advancements into 3D imaging techniques have facilitated volumetric imaging capabilities not previously available on an *in vivo* basis; however, accurate dental volume measurement procedures are required in order to fully utilize the technology. The resulting use of 3D imaging has enabled the quantification and measurement of ERR to be completed with a high level of diagnostic accuracy and repeatability when compared to periapical radiographs [9,16-18]. The strength of cone-beam computed tomography (CBCT) for accurate dental volume measurements *in vivo* has been shown not to be statistically significantly different as *in vitro* measurements in one study [19] and even when comparing its accuracy to *in vitro* micro-CT imaging methods [20]; however, there may exist machine-specific variations. The feasibility of *in vivo* dental volume measurements using CBCT imaging has similarly been reported by Liu et al*.*; however, their use of post-processing surface smoothing has been shown to decrease 3D volume measurements [21]. Conversely, increasing the voxel size has been shown *in vitro* to actually increase volume measurements [22]. It is intuitively apparent that the accuracy of the 3D segmentation procedure is related to the voxel size during acquisition [23] with 0.25 mm voxel size an appropriate compromise between diagnostic accuracy and patient radiation dose [22]. An additional factor is the development of a clearly defined measurement protocol, which appears lacking in the literature as the study currently employing CBCT as a means of determining root volume loss with maxillary expansion lacks a clearly defined measurement protocol involving incorrectly utilized Hounsfield units with the teeth of interest ‘segmented cautiously’ [24]. There exists a potential limiting factor inherent in the use of CBCT scans to measure accurate volumetric information as the time period required to capture the radiograph as patient movement during scans can reduce the accuracy of measurements [15,25].

The validation of CBCT as a tool for measurement of both root lengths and volumes has been focused on in numerous studies [15-17,19,21,26-33]. The investigation of *in vivo* volumetric determination utilizing CBCT images has been shown to yield slight differences from actual physical volumes within −4% to +7% [21]. However, there is a lack of a clearly defined gold-standard 3D segmentation protocol in the literature.

Given the inconsistencies of the techniques reported in the literature, the development of an appropriate CBCT measurement protocol possessing accuracy and precision in both intra- and inter-rater reliability for *in vivo* dental volume measurements is desired. Due to the relative infancy of the area of 3D dental volume segmentation, with a lack of a gold-standard technique, the need to employ and evaluate segmentation techniques to identify which measurement protocol is most superior is a necessity. Through the establishment of precise and accurate dental volume measurement protocols, clinicians can more confidently employ the available tools to monitor such phenomena as ERR during the orthodontic process and ERR related to dental trauma.

The objective of this study is therefore to analyze a set of developed and proposed image segmentation protocols for precision in both intra- and inter-rater reliability for *in vivo* tooth volume measurements using CBCT images.

## Methods

### Cone-beam CT images

The radiographic data set used for the analysis of dental volume was previously acquired as part of a randomized clinical trial at the University of Alberta, Edmonton, Alberta, Canada. Subjects were recruited during an 18-month period. Inclusion criteria for selection included transverse maxillary deficient adolescents with no previous orthodontic treatment. The age range of patients selected for this study ranged from 11 to 17 years old. Subjects were not excluded based on the presence or absence of coronal restorations. Informed consent from the patients' parents and ethical approval from the Ethics Committee at University of Alberta was obtained.

All CBCT images were taken with the NewTom 3G (QR SRL, Verona, Italy) device at 110 kV, 6.19 mAs, and 8-mm aluminum filtration with the patient in maximum intercuspation following common CBCT imaging protocols. Images were converted to DICOM format by using the NewTom software to a voxel size of 0.25 mm. The DICOM-formatted images were volume rendered with Avizo 3D analysis software (Visualization Sciences Group, Berlin, Germany) [34]. Patient images were acquired at two time points during the trial: T1 (before treatment) and T2 (after treatment, approximately 12 months).

### Tooth volume measurement protocols

Three protocols for dental tooth volume determination were investigated using Avizo 3D analysis software:

1. Manual human segmentation on a repeated 2D basis;
2. Automated segmentation without human refinement; and
3. Automated segmentation with manual human refinement on a repeated 2D basis.

In addition, two methods for tooth volume selection were simultaneously investigated. These involved the entire tooth structure including the crown and only the dental root structure apical to the cemento-enamel junction (CEJ). All three protocols and two methods were applied to determine the technique producing greatest intra- and inter-rater reliability. The dental pulp chamber and canals were included in the volume measurements. The investigator was blinded to whether they were T1 (before treatment) or T2 (after treatment) radiographs. In all, a total of six different approaches (combination of three protocols and two tooth volumes) to tooth volume segmentation were investigated. Ten randomly selected maxillary first molars (selected from both T1 and T2 patient images) were measured *in* vivo in random order three times with 10 days separation between measurements.

The threshold value for image segmentation was set for each tooth. This same threshold value was used in all protocols to assess the particular tooth of interest to limit variability between methods. The first protocol did not require a threshold value to be explicitly set as the protocol was strictly manual human tracing of the image on a 2D slice-by-slice basis.

No image orientation adjustments were completed prior to testing of the protocols. The sagittal plane was utilized for initial evaluations for each technique, as it appeared most useful in the visualization and evaluation of the tooth structure of the crown and root simultaneously.

**Figure 1:**
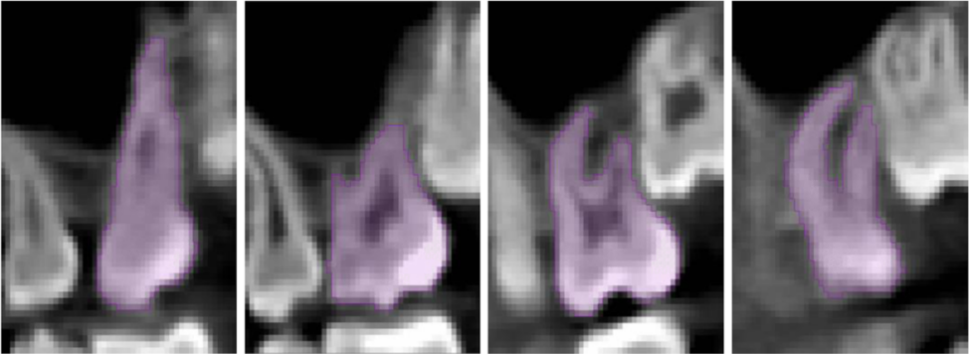
**YZ (sagittal) plane.**

**Figure 2:**
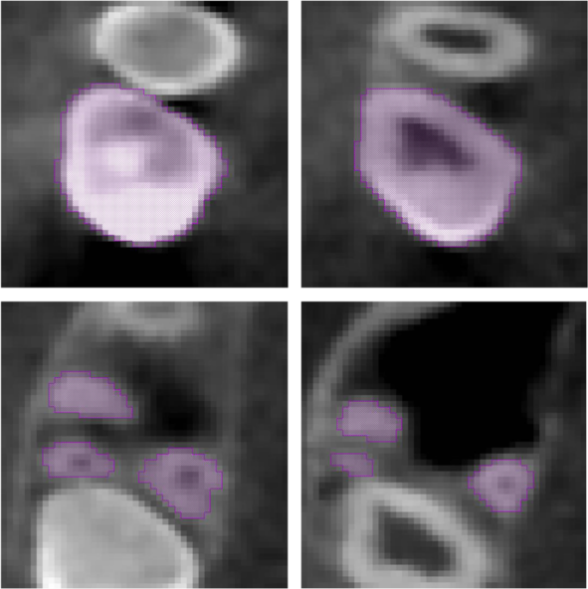
**XY (axial) plane.**

**Figure 3:**
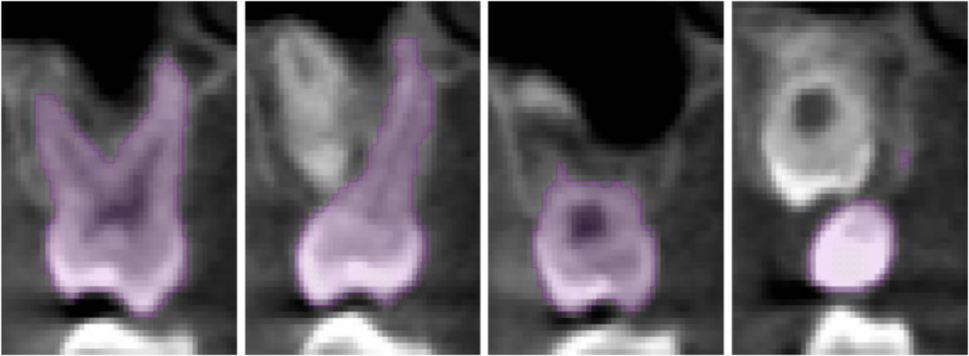
**XZ (coronal) plane.**

**Figure 4:**
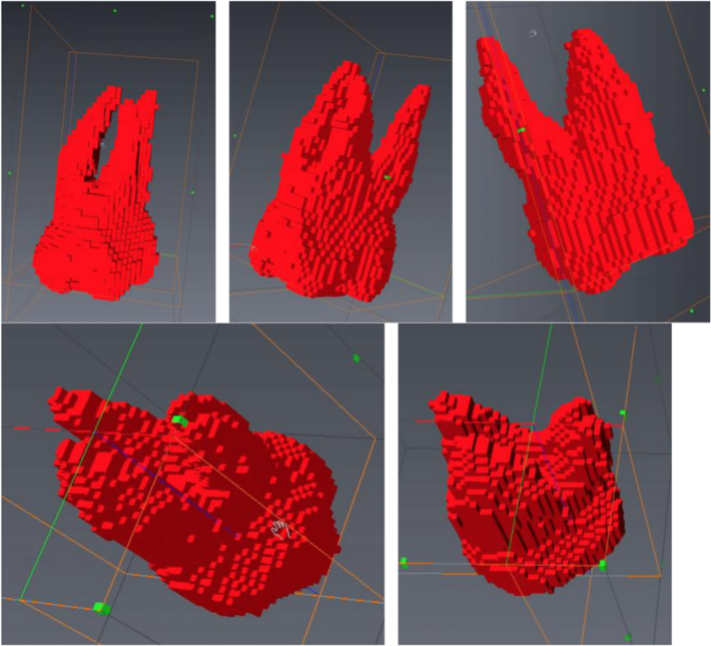
**3D sample view of maxillary first molar volume without smoothing.**

**Figure 5:**
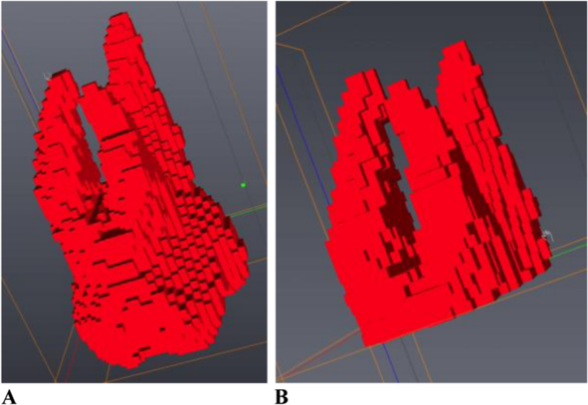
**Three-dimensional tooth volume.** **(A)** Three-dimensional complete tooth volume. **(B)** Three-dimensional tooth volume apical to the cemento-enamel junction.

- 1. Manual human segmentation on a repeated 2D basis
- The first protocol involved manual image segmentation procedures on a 2D slice-by-slice basis through the use of Avizo's ‘lasso’ tool, which allows one to define an area freehand by generating a closed contour curve in 2D. The delineation of tooth structure from the surrounding alveolar and cortical bone was first determined on a slice-by-slice basis in the YZ (sagittal) plane (Figure 1) based upon visual inspection only. Refinements in the XY (axial) plane (Figure 2) were then manually completed for the observation of the tooth anatomy from a different perspective. An axial view enabled root structure and interproximal contact point refinements. Finally, additional refinements in the XZ (coronal) plane (Figure 3) were again manually completed. A coronal view enabled refinements to root structure that was in close proximity to the buccal and palatal cortical plates. The 3D resultant tooth was evaluated for approximately normal maxillary first molar dental anatomy to limit gross misidentification of dental structures (Figure 4). Once segmentation was completed, the software automatically computed the tooth's radiographic volume. No smoothing functions were applied to the 3D tooth structure to prevent smoothing of minor root defects/imperfections or possible resorption lacunae. Both the complete tooth volume (Figure 5A) and the dental root volume, defined as the anatomical root apical to the CEJ, (Figure 5B) were measured.
- 2. Automated segmentation without human refinement
- The second protocol involved the use of the ‘magic wand’ tool in Avizo 3D imaging software as a ‘region-growing’ tool. The ‘magic wand’ tool allows one to perform the so-called ‘region-growing’ in either 2D or 3D. Selecting an individual ‘seed voxel’ of a tooth root or crown selects the largest connected area (either 2D or 3D) that contains the voxel itself and all voxels with gray values contained within a user-specified range. The range can be chosen to represent absolute gray values or gray values relative to that of the seed voxel. For the purposes of our investigation, absolute gray values were chosen to limit variability in selection of the seed voxel gray value. Segmentation was performed using strictly an automated approach after minor operator input to the selection of the seed voxel in the enamel of the tooth without focused manual refinements in an attempt to test an efficient measurement procedure. The user input to select the seed voxel proved to be a necessity given the software. The rest of the procedure required no operator input for the actual segmentation procedure. A visually defined optimal threshold value was set for each tooth in the YZ (sagittal) plane (Figure 1). The threshold level was set to most clearly show the tooth anatomy with minimal interference from the surrounding bone and adjacent structures. The 3D resultant tooth was evaluated for approximately normal maxillary first molar dental anatomy to limit gross misidentification of dental structures. Once segmentation was completed, the software automatically computed the tooth's radiographic volume. As in protocol 1, no smoothing functions were applied and both the complete tooth volume and dental root volume were measured.
- 3. Automated segmentation with manual human refinement on a repeated 2D basis
- The third protocol also involved the use of the ‘magic wand’ tool in Avizo 3D imaging software as a ‘region-growing’ tool, similar to that utilized in the second protocol; however, in this case, segmentation was performed using a mixture of an automated approach with manual localized visual refinements to the tooth structure. For the purposes of our investigation, absolute gray values were chosen to limit variability in selection of the seed voxel gray value. The same absolute gray value range was selected as in the second protocol to limit variability between methods for each tooth. Segmentation was performed using a mixture of an automated approach with manual localized visual refinements to the tooth structure. A visually defined optimal threshold value was set for each tooth in the YZ (sagittal) plane (Figure 1). The threshold level was set to most clearly show the tooth anatomy with minimal interference from the surrounding bone and adjacent structures. Manual refinements were processed on a slice-by-slice basis to enhance accuracy by correcting for over- and under-contoured voxels in the tooth volume. Initial refinements occurred in the YZ (sagittal) plane. Secondary refinements were performed in the XY (axial) plane (Figure 2) to refine root structure and interproximal dental contact points. Tertiary refinements were performed in the XZ (coronal) plane (Figure 3) to verify tooth anatomy and focus on the delineation of dental root structure from the buccal and palatal cortical plates. The 3D resultant tooth was evaluated for approximately normal maxillary first molar dental anatomy to limit gross misidentification of dental structures. Once segmentation was completed, the software automatically computed the tooth's radiographic volume. As in protocols 1 and 2, no smoothing functions were applied and both the complete tooth volume and dental root volume were measured.

#### Statistical analysis

The volume data was manually entered into Microsoft Excel 2011 for MAC (Microsoft, Redmond, WA, USA). SPSS for MAC (version 21, IBM, Armonk, New York, USA) was used to run all statistical tests. For all tests, statistical significance was set at an *α* value of 0.05.

Intra-class correlation coefficient (ICC) was used to measure the agreement between the measurements for the continuous dependent variable (dental tooth volume) taken on the three separate days. A single measure with consistency under two-way mixed model was chosen, thus removing the rater's variation, and the subjects/teeth were chosen randomly with the rater fixed. The technique that produced the highest ICC value and lower bound of the 95% confidence interval was chosen as the preferred measurement protocol.

To assess inter-rater reliability for the two approaches, the second rater (S.N.) was trained directly by the initial rater step-by-step in the use of the software and chosen measurement technique as determined from the intra-examiner reliability assessment. The general use of the software, visualization of the tooth of interest in all three planes of space, automated segmentation procedures, manual refinements, and 3D visualization of the resultant volume were reviewed in training. The second rater (S.N.), who possessed a dental background and knowledge of normal dental anatomy, measured the same ten randomly selected maxillary first molars as measured by the principal investigator (both the whole tooth method and the dental root apical to the CEJ method). ICC was used to measure the agreement between the principal investigator's second measurement, as determined randomly, and the additional investigator's single measurement. A single measure with absolute agreement under two-way mixed model was chosen to account for rater variation, and the subjects/teeth were chosen randomly with the raters fixed.

## Results

### Intra-rater reliability

- Protocol 1: manual human segmentation on a repeated 2D basis
- The ICC demonstrated agreement, ICC (single measures) = 0.885, 95% CI (0.707, 0.967), within rater for the whole tooth measurement. The ICC demonstrated agreement, ICC (single measures) = 0.904, 95% CI (0.749, 0.973), within rater for the root measurement apical to the CEJ.
- Protocol 2: automated segmentation without human refinement
- The ICC demonstrated agreement, ICC (single measures) = 0.826, 95% CI (0.697, 0.952), within rater for the whole tooth measurement. The ICC demonstrated agreement, ICC (single measures) = 0.899, 95% CI (0.742, 0.953), within rater for the root measurement apical to the CEJ.
- Protocol 3: automated segmentation with manual human refinement on a repeated 2D basis
- The ICC demonstrated excellent agreement, ICC (single measures) = 0.996, 95% CI (0.989, 0.999), within rater for the whole tooth measurement. The ICC demonstrated agreement, ICC (single measures) = 0.904, 95% CI (0.751, 0.973), within rater for the root measurement apical to the CEJ.

Therefore, the whole tooth measurement utilizing protocol 3 was selected as the measurement method as it possessed the highest ICC value and lower bound of the confidence interval (ICC (single measures) = 0.996, 95% CI (0.989, 0.999)) when compared to all other measurement protocols investigated.

The summary of intra-rater reliability via the ICC is presented in Table 1. In addition, a summary of the largest volume differences for intra-rater repeated measures is presented in Table 2.

**Table 1:** **Intra-rater intra-class correlation coefficient values**

**Measurement protocol**	**Volume measured**	**ICC**	**CI (lower bound)**	**CI (upper bound)**	***F*** **-text,** ***p*** **values**
Protocol 1: manual	Whole tooth	0.885	0.707	0.967	*F* (9,18) = 24.158, *p* < .0005
Protocol 1: manual	Roots apical to CEJ	0.904	0.749	0.973	*F* (9,18) = 29.406, *p* < .0005
Protocol 2: automated	Whole tooth	0.826	0.697	0.952	*F* (9,18) = 12.215, *p* < .0005
Protocol 2: automated	Roots apical to CEJ	0.899	0.742	0.953	*F* (9,18) = 27.512, *p* < .0005
Protocol 3: automated with refinements	Whole tooth	0.996	0.989	0.999	*F* (9,18) = 767.557, *p* < .0005
Protocol 3: automated with refinements	Roots apical to CEJ	0.904	0.751	0.973	*F* (9,18) = 29.406, *p* < .0005

CEJ = cemento-enamel junction; CI = confidence interval; ICC = intra-class correlation coefficient.

**Table 2:** **Largest volume differences for intra-rater repeated measures**

	**Volume measured**	**Largest difference (single rater) (mm** ^**3**^ **)**
Protocol 1:	Whole tooth	49.15
	Roots apical to CEJ	76.21
Protocol 2:	Whole tooth	52.51
	Roots apical to CEJ	75.15
Protocol 3:	Whole tooth	17.76
	Roots apical to CEJ	64.79

CEJ = cemento-enamel junction.

### Inter-rater reliability

Looking strictly at the variability on an intra-rater basis was the focus of our determination of appropriate methods to be evaluated on an inter-rater basis. Therefore, the method with the highest intra-rater reliability was chosen to further address inter-rater reliability. Given that protocol 3, automated segmentation with manual human refinement on a repeated 2D basis, yielded the highest intra-rater reliability statistics, the inter-rater reliability was computed utilizing measurement protocol 3. The ICC demonstrates excellent agreement, ICC (single measures) = 0.990, 95% CI (0.961, 0.998), between raters for the whole tooth measurement. However, the ICC demonstrates less powerful agreement, ICC (single measures) = 0.728, 95% CI (0.198, 0.926), between raters for the root measurement apical to the CEJ. The inter-rater analysis results are in agreement with the chosen measurement protocol as determined via intra-rater ICC values.

The reliability readings for protocol 3 are included in Table 3. It serves to display the differences in absolute volume measurements for the repeated measures and inter-rater values.

**Table 3:** **Reliability readings for protocol 3 (all units in mm** ^**3**^ **)**

	**Rater-first measurement**	**Rater-second measurement**	**Rater-third measurement**	**Rater 1 average measurement**	**Rater-1 largest volume difference measurement**	**Rater 2 measurement**	**Rater 1 average versus rater 2 difference**	**Rater 1 (second measurement) versus rater 2 difference**
Whole tooth	1,072.26	1,058	1,063.25	1,064.50	14.26	1,056.25	8.25	1.75
	1,054.7	1,066.26	1,060.58	1,060.51	11.56	1,077.73	17.22	11.47
	1,019.74	1,030.25	1,033.55	1,027.85	13.81	1,039.89	12.04	9.64
	1,017.71	1,020	1,021.32	1,019.68	3.61	1,006.65	13.03	13.35
	990.74	992.42	993.24	992.13	2.5	1,005.58	13.45	13.16
	1,195.89	1,178.13	1,170.26	1,181.43	17.76	1,160.21	21.22	17.92
	972.53	968.71	965.85	969.03	6.68	995.22	26.19	26.51
	859.34	851.43	853.25	854.67	7.91	838.88	15.79	12.55
	1,229.75	1,219.06	1,221.39	1,223.40	10.69	1,242.85	19.45	23.79
	1,251.74	1,238.41	1,249.85	1,246.67	13.33	1,216.28	30.39	22.13
Roots apical to CEJ	589.15	552.04	542.03	561.07	47.12	601.54	40.47	49.5
	499.17	505.27	489.25	497.90	16.02	546.83	48.93	41.56
	546.6	532.21	525.24	534.68	21.36	500.24	34.44	31.97
	452.29	487.68	442.88	460.95	44.8	402.86	58.09	84.82
	607.23	607.89	615.25	610.12	8.02	548.95	61.17	58.94
	610.79	657.77	662.66	643.74	51.87	630.84	12.90	26.93
	504.22	533	492.02	509.75	40.95	552.21	42.46	19.21
	403.67	445.49	394.12	414.43	51.37	459.21	44.78	13.72
	498.52	490.25	483.66	490.81	14.86	480.65	10.16	9.6
	578.15	527.26	592.05	565.82	64.79	607.54	41.72	80.28

CEJ = cemento-enamel junction.

One subject had coronal restorative material present in the evaluated tooth. The presence of this restorative material did not have significant effects on the segmentation results, as it was not an outlier in the data set.

## Discussion

The method involving automated segmentation with manual human refinement on a repeated 2D basis for the whole tooth proved to be the most reliable measurement protocol both within and between observers. Essentially, the intra- and inter-rater analysis results are in agreement with measurement protocol 3 as determined via ICC values. For excellent agreement, the ICC 95% confidence interval should be above 0.750 [35,36], which is the case for the results obtained for protocol 3 using the entire tooth volume. It is of value to note that the protocol developed possesses similarities to studies investigating the accuracy of dental volume measurement in vivo using CBCT [21] and condylar head volume measurement [37], and hence lends to our segmentation technique's credibility.

The greatest difference across intra-rater repeated measures for the whole tooth approach utilizing protocol 3 was 17.76 mm^3^ (approximately 1.50% of the average whole tooth volume measured), whereas for the roots, only approach utilizing protocol 3 was 64.79 mm^3^ (approximately 11.45% of the average root volume apical to the CEJ measured). The intra-rater variability was thus approximately 3.6 times greater in absolute volume (64.79 versus 17.76 mm^3^) and 7.6 times greater in proportion of structure measured (11.45% versus 1.50%) when measuring roots only as compared to measuring the entire tooth volume.

**Figure 6:**
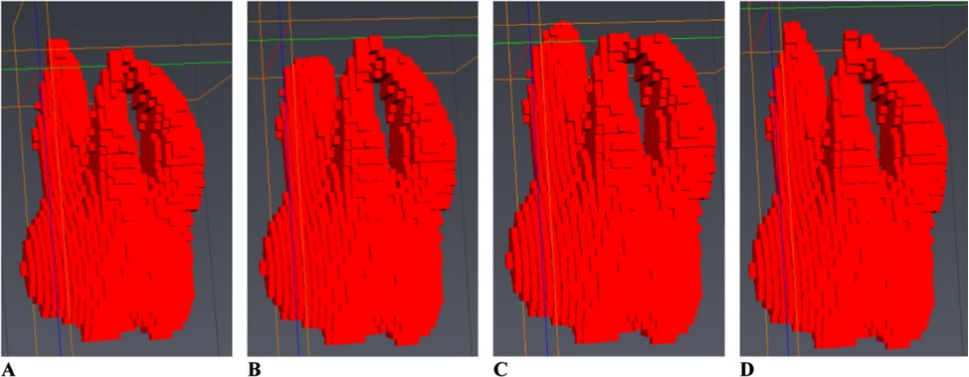
**Three-dimensional visualization of inter-observer volume differences for the whole tooth measurement. (A)** Entire tooth volume. **(B)** Entire tooth volume with maximum inter-observer variability removed from palatal root. **(C)** Entire tooth volume with maximum inter-observer variability removed from apical portion of all three roots. **(D)** Entire tooth volume with maximum inter-observer variability removed from buccal surfaces of the mesiobuccal and distobuccal roots.

**Figure 7:**
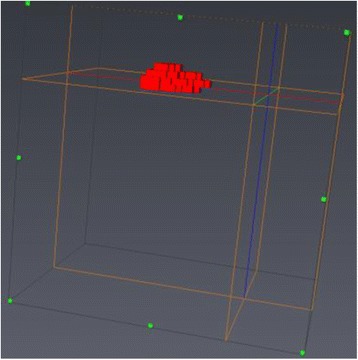
**Three-dimensional visualization of the intra-observer volume difference for the maxillary right first molar mesio-buccal cusp tip.**

The visualization of the respective maximum volume difference as displayed in Table 3 applied to a single tooth in various scenarios is displayed in Figure 6. The maximum volumetric discrepancy between repeated intra-observer measurements for the whole tooth was 17.76 mm^3^. The maximum volumetric discrepancy for inter-observer measurements for the whole tooth was 30.39 mm^3^. The effects of these measurement variations can be shown visually in a number of ways as displayed in Figure 6. Removal of the maximum inter-observer volume difference (30.39 mm^3^) strictly from the most apical portion of the palatal root (Figure 6B), from the apical portions of all three roots (Figure 6C), and from the buccal surfaces of the mesiobuccal and distobuccal roots are displayed (Figure 6D). Nearly imperceptible changes when differences are distributed across all roots and on the buccal surfaces of the mesiobuccal and distobuccal roots visually display the inter-observer errors with which tooth volumes may be determined. Visualizing the maximum volumetric discrepancy between repeated intra-observer measurements for the whole tooth of 17.76 mm^3^ is displayed visually in Figure 7. The volume displayed is the mesiobuccal cusp tip of the maxillary right first molar. The occlusal-apical dimension of the cusp tip volume measures only 1.2 mm, thus providing an approximate clinical interpretation and visualization of the volume differences. In addition, when considering the measurements of ERR with tooth-anchored maxillary expander (TAME) for the maxillary first molars completed by one rater, average maxillary first molar ERR volume changes of 42.67 mm^3^ have been previously reported in the literature [24], which is approximately 2.4 times greater than the intra-rater reliability protocol established for this technique.

The method resulting in the worst reliability was automated segmentation without human refinement. There are numerous reasons why this protocol was flawed. The determination of the boundaries between the tooth roots and the buccal and palatal cortical plates is sometimes indistinct given the very close proximity of the roots. The furcation area of the tooth possesses a large surface area of lamina dura, the dense surrounding cortical bone, which can lead to unclear tooth furcation anatomy. The proximity of the erupting second molar in some patients, as well as the interproximal contacts with adjacent teeth, often led to over-contouring of the volume of the crown of the maxillary first molar. In addition, the presence of dense bone islands of increased radiopacity can also result in misidentification of the proper root morphology. Given the limitations associated with a strictly automated method, it is still not possible, at least at a 0.25 mm voxel size, to automate the segmentation procedure. To be precise, the process must still involve manual human refinements with proper knowledge and interpretation of the 3D anatomy. As such, the process is extremely labor intensive given the slice-by-slice refinements that are required in all three planes of space.

In the approaches investigated, the pulpal tissue was included in the volumes as additional errors in delineating dentin from pulpal tissue would be an added source of variation. The additional dentin/soft tissue border, which is likely more challenging anatomy than the tooth to surrounding bony support to identify, due to intricate pulpal canal architecture of small dimension, would require identification. Therefore, since our area of interest is only ERR, internal pulpal changes are irrelevant. Consequently, both the hard and soft tissues within the cementum of the tooth were calculated as a part of the total tooth volume.

Due to the retrospective nature of the study, certain limitations were imposed on our ability to verify the accuracy of the volume measurements. To address this concern, focus was turned to the precision of volume measurements from both an intra- and inter-examiner perspective. The limitations from the retrospective nature of the study are twofold. Firstly, the CBCT machine used to capture the initial images was no longer functional or available for additional measurements such as *in vitro* dental volume comparisons. Secondly, due to the non-extraction orthodontic treatment of these patients, and that investigation of maxillary first molar volume was desired, the true value of the molar volumes is unknown and is unlikely to be known in future studies as maxillary first molars are not commonly extracted for orthodontic purposes except in rare circumstances. However, there appears no obvious reason for not being able to extrapolate the measurement protocol identified to other teeth. Therefore, with the inability to focus on the validity of the data, the approach was chosen to identify a measurement protocol to give highly precise results, both in intra- and inter-rater conditions.

The desire and ability to verify the true volumes or accuracy for this particular CBCT machine brings into question the capability to replicate an *in vivo* scenario. Numerous factors could not be addressed in a *post hoc* replicated model including the lack of a periodontal ligament, cortical bone, soft tissues, and patient movement to name a few. In addition, the imaging of a model as opposed to an *in vivo* dental volume followed by extraction and *in vitro* dental volume measurement would lead to the introduction of several errors and inaccuracies.

The validation of CBCT as a tool for measurement of both root lengths and volumes has been addressed in a number of studies with a multitude of image segmentation techniques. [16,17,19-21,27-33] The weaknesses of the studies include the lack of investigation into more than one image segmentation protocol to provide the most precise experimental data. As an example, the study assessing ERR with maxillary expansion using 3D CBCT images [24] utilizes a segmentation procedure employing a root-only approach (apical to the CEJ for maxillary premolars and apical to the deepest point of the furcation for maxillary molars). The results of our study yielded the greatest intra-rater variability when using a similar method. Therefore, assessment of the root volume only appears to be wrought with errors in identification of the desired volume. Although an identical CBCT machine was not utilized in our own study, the voxel size and imaging parameters were similar to that of another [21]. In general, a change of software or CBCT machine appears to not be significantly clinically important given voxel sizes are held constant. There exist other 3D software programs for analyzing CBCT data with similar functions as the software is being utilized only as a tool to compute a volume. What does appear important however is voxel size and segmentation protocol, not the particular software used, as long as there is segmentation functionality.

Due to the uniqueness of our data set and limited access to the original CBCT machine because of the retrospective nature of the study, validation of our method, was sought in the literature. After the independent development, reliability testing, and subsequent comparison with existing published literature employing image segmentation protocols for dental volumes, some conclusions were reached. With numerous segmentation protocols in the literature, the volume measurement techniques in one CBCT volume validation study [21] were identical (in so far as can be determined from their reported methods) to our protocol 3 (whole tooth), which possessed the most precise volume segmentation results. Given the similarity of our measurement protocols, we feel confident in the validity of our results obtained to the study that verified the accuracy and validity of the dental tooth volume to within −4% to 7% [21].

Traditionally, as reported in the literature, bicuspids were routinely measured *in vivo* and subsequently extracted for physical volumetric determination [21]. There is an inherent tooth type limitation likely to be present in all studies due to the rarity of maxillary first molar extractions associated with orthodontic treatment. In our study, investigation of the maxillary first molar was chosen for a number of reasons including its complex root anatomy, early eruption and completion of root development in orthodontic-aged adolescent patients, and its use as an anchorage unit for initial phase orthodontic care. The potential for incomplete root development would be a limitation in evaluating any permanent tooth in adolescents, but given the comparatively early eruption of the permanent first molars, this limitation is mitigated as much as possible. An additional reason for this decision is because utilizing CBCT to assess ERR associated with maxillary expansion appliances is the ultimate goal. With maxillary first molars being the most commonly banded teeth that are attached to a maxillary expansion appliance, it makes inherent sense to assess the resorptive changes occurring within the anchor teeth themselves.

**Figure 8:**
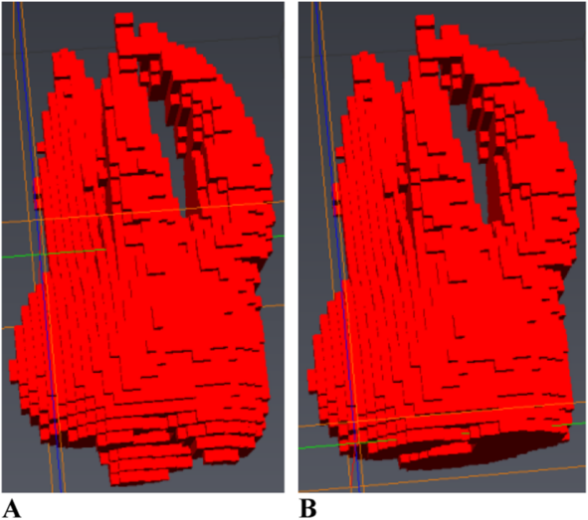
**Cusp tip attrition.** This would have to be present to represent the maximum difference in additional variability between roots only and whole tooth in the repeated measures. **(A)** Normal 3D volume. **(B)** 3D volume representing maximum difference in additional variability of the roots versus the whole tooth (64.79 mm^3^ − 30.39 mm^3^ = 34.40 mm^3^) in the repeated measures removed from cusp tips.

**Figure 9:**
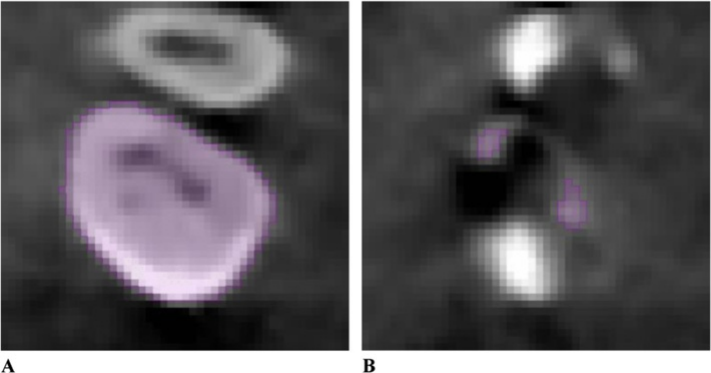
**Axial slice area at the cement-enamel junction (CEJ) versus cusp tip. (A)** Axial slice area at the molar CEJ. **(B)** Axial slice area at the molar cusp tips.

A strength of our reliability investigation lies not only in the numerous image measurement protocols investigated but also in the investigation of the entire maxillary first molar tooth volume versus the volume of the roots apical to the CEJ for each protocol as different segmentation cutoff points have been reported in the literature with no justification [24] or mention of technique accuracy or reproducibility. The reason two volumes were investigated is numerous. If the entire tooth volume was used, this adds the potential for patient coronal tooth volume variability between time points due to possible attrition, decay, loss of coronal tooth structure, and the placement or adjustment of new fillings or occlusion, in addition to other unidentified sources. This coronal tooth structure variability was not directly investigated in this particular paper, as the volume measurements for reliability were repeated measures on the same teeth at the same point in time. However, the identification and attention to the possible sources of variability aids in deciding whether the variability present in the root volume only measurements apical to the CEJ approach is more favorable due to the absence of the coronal variability. To address this from a visual perspective, Figure 8 displays the coronal changes that would have to be present on the cusp tips of the maxillary first molars to represent the maximum difference in additional variability of the root versus whole tooth (64.79 mm^3^ − 30.39 mm^3^ = 34.40 mm^3^) in the repeated measures. With visualization of the hypothetical coronal changes, it appears clear that measuring the whole tooth appears superior when compared to the roots only given the additional variability associated with measuring the roots only is more than can be expected from coronal changes over the short term (1 to 2 years). Support for the use of the whole tooth measurement protocol comes from a study exploring crown and root length of teeth using CBCT images. The study found a wider range of limits, and hence more variability, in measuring root lengths as opposed to crown lengths [26]. This increased error can perhaps be extended to root volumes due to difficulties in determining the CEJ location as the enamel is at its thinnest in this area. The CEJ is anatomically not a straight line; however, in many images, the lack of definition of the apical extent of the enamel resulted in a nearly straight-line resultant 3D segmentation. The best suggested method considers all the tooth structure not just the root in order to eliminate CEJ identification. For instance, an error in identification of one axial 2D slice was found to introduce root volume changes between 40 to 65 mm^3^ depending on tooth size. The increased variability appears to occur due to the fact that the CEJ represents quite a large 2D axial volume. In contrast, attrition at the molar cusp tips leads to almost imperceptible changes in tooth volume. The differences between a cusp tip axial slice area versus a CEJ slice area are displayed in Figure 9. Therefore, due to the increased difficulties in CEJ identification, the role of external coronal volume changes from T1 to T2 were judged to be minimal compared to the effects of an error in CEJ identification.

In general, the presence of radiodense restorative materials, such as amalgam and some highly filled composites, which greatly inhibit the passage of electromagnetic radiation, has the potential to introduce further variability into dental volume determination and result in imaging artifacts. These artifacts can result in the inability to predictably identify the true extent of the radiodense material or adjacent structures and thus affect volume segmentation.

There exists the obvious issue of resolution of a CBCT image in determining the volume of a tooth. Given the voxel size of 0.25 mm in each dimension, there are concerns regarding the potential that the border of a tooth versus bone could be contained within a voxel. A limitation in computed tomography imaging is the so-called ‘partial volume effect.’ In essence, this phenomenon can present issues in the differentiation of different tissue types. For example, a large amount of periodontal ligament (PDL) space and a thin layer of compact bone such as the lamina dura can cause the same attenuation in a voxel as the dentin of a tooth alone. The issue of resolution is complex as improved resolution can be acquired, but at the expense of increased patient radiation dose [30]. Although improved volumetric determination and ERR detection can be obtained with smaller voxel sizes and increased scan times [28], there exists a limit, which has to be established between patient radiation dose and resolution required for appropriate diagnostics. However, early detection of root changes may modify treatment mechanics and thus limit the progression of ERR and the long-term impact on the affected teeth. Using a voxel size of 0.125 mm has the potential to yield *in vivo* volume measurement of teeth comparable to micro-CT *in vitro* analysis [20], but understandably has the disadvantage of increased patient radiation dose. A study, which investigated the influence of voxel size on the diagnostic ability of CBCT to evaluate ERR, concluded CBCT to be a reliable method of ERR detection with a voxel size of 0.3 mm as the ‘best protocol’ when balancing patient dose and diagnostic performance [38]. Given the voxel size of 0.25 mm used in this study, there appears to be more than adequate resolution required to measure dental tooth volumes while balancing patient radiation dose.

A limitation of the study involves the use of only one CBCT model to capture the patient images. There exists the potential issue of variation between different CBCT models that may possibly possess varying image quality and gray value distributions for the aforementioned segmentation methods.

Through the establishment of precise and accurate dental volume measurement protocols, clinicians can more confidently employ the available tools to monitor such phenomena as ERR at various stages throughout the orthodontic process and ERR related to dental trauma. However, patient radiation exposure and diagnostic imaging needs require a careful balance to be established. The uses of CBCT imaging are to maximize the diagnostic information available to the clinician while limiting patient radiation exposure to make individualized treatment decisions while considering as many patient specific factors as possible.

## Conclusions

The proposed maxillary first molar dental volume measurement protocol for CBCT images employing automated segmentation with manual human refinement on a repeated 2D slice-by-slice basis in all three planes of space possesses excellent intra- and inter-rater reliability and precision.

Maxillary first molar 3D volume measurements of the entire tooth structure are more precise than 3D volume measurements of only the dental roots apical to the CEJ.
